# Samuel Henry Swayne

**Published:** 1900-12

**Authors:** 


### Obituary.

### SAMUEL HENRY SWAYNE.

We have to record, with deep regret, the death of Mr. Samuel Henry Swayne in his eightieth year.

His was a well-known figure at the meetings of the Bath and Bristol Branch of the British Medical Association. There, as also at the Bristol Medico-Chirurgical Society, he was always listened to with interest, from the fact that he spoke only of matters with which he was thoroughly conversant. A member of an old Gloucestershire family, he was born in Bristol, and spent his whole professional life here. He owed his early medical education partly to the Bristol Medical School, and partly to St Bartholomew's in London. Much of his student experience was gained in the wards of the Bristol Royal Infirmary.

Never in robust health, he carried on a useful practice, and was much valued by his patients. His honourable dealing in all professional matters raised for him a deep respect amongst his medical brethren; and in any discussion on scientific matters in which he took part, it was felt that he brought to bear on the subject in hand the result of extensive scientific reading and research. His pure, good life teaches many lessons. No one ever knew him without feeling a real regard for him. In quietness and in confidence was his strength; and it is not a usual record that he was in practice within a few weeks of his death, and that he found opportunities of interest, not only in the purely medical societies of the district, over one of which, the Medico-Chirurgical, he had some years ago presided, but also in the Bristol Naturalist Society, the Bristol and Gloucester Archæological Society, the Bristol Microscopical Society, and the Clifton Antiquarian Club.

He was a tower of strength in friendship, and a deeply religious man. *Requiescat in pace.*

